# A New Method for Publishing Three-Dimensional Content

**DOI:** 10.1371/journal.pone.0007394

**Published:** 2009-10-20

**Authors:** Eugene Raush, Max Totrov, Brian D. Marsden, Ruben Abagyan

**Affiliations:** 1 Molsoft LLC, La Jolla, San Diego, California, United States of America; 2 Nuffield Department of Clinical Medicine and the SGC, University of Oxford, Headington, Oxford, United Kingdom; University of Cambridge, United Kingdom

A new method for electronic publishing of articles with text linked to its interactive three dimensional content is described. The method is based on a single document containing a variety of objects such as formatted text, multiple three dimensional molecular objects, textured shapes and surfaces, data tables and graphs, chemical spreadsheets, alignments, etc. The 3D article can then be published for an online web delivery using the activeICM/active X components as well as be downloaded as a single file to be browsed with all its attached objects locally with the ICM browser. Both activeICM and ICM browser are freely available for the public. This method eliminates the need for multiple methods for the web and the local off-line delivery; it offers the dramatically enhanced, customizable and interactive delivery of article's three dimensional content and data attachments in a single compact file.

## Introduction

The common approach to electronic publishing three-dimensional biological data in peer-reviewed journals or for disseminating such data within the scientific community has historically required the writing of a two dimensional flat and non-interactive document. The frequent limit on the number of two dimensional color figures permitted in journals further reduces the opportunities for the provision of visualizations. Often the targeted readers of these documents are not expert in the gathering and manipulation of the fundamental data from which the document was provided. For example, three-dimensional protein structures and complexes require the downloading of coordinates from the PDB [1] and then the knowledge and skill to operate a standalone program to simply reproduce the view corresponding to the figure in question and to further interpret it. Further data, such as protein sequences, parts of the electron density [2], etc., may also need to be obtained from remote databases and manipulated to interpret the results and conclusions of the underlying publication. This approach is clearly sub-optimal for scientists who have no experience in protein structure and its visualization.

A breakthrough in the ability to collate and integrate pertinent data types and annotations relating to such data into one file that is compact and platform-independent was the iSee platform [3]. In this approach, arbitrary numbers of three-dimensional structural visualizations are possible and the renditions are embedded via hyperlinks in a textual annotation akin to a standard scientific publication. Previously these so-called iSee ‘datapacks’ could be read and viewed using freely-available browser software. A number of other approaches to visualize three-dimensional structures within the context of text have been made since iSee was developed (e.g. [4]–[6]). However, neither these nor iSee have been able to provide full integration within the requirements of peer-reviewed publications and to support both the web delivery of an electronic publication and the local file delivery in a consistent manner. We have recognized that there is a clear requirement to improve the integration and delivery for publication and general dissemination of this type of work.

In this overview we provide details of our activeICM approach for the peer-reviewed publishing and sharing of integrated and interactive iSee datapacks, or 3D documents, which is the core technology of the PLoS ONE Collection of which this overview paper is part of.

## The Publication Method

The activeICM method is able to represent three-dimensional documents, such as iSee datapacks, in a manner independent of hardware and operating system and web-accessibility using only freely-available components. The method is based on several key elements, some of which stem from the original single document with the stored views and links concept previously presented [3], and some of which evolved as a result of the Web and Microsoft Office document delivery paradigms.

**Figure 1:**
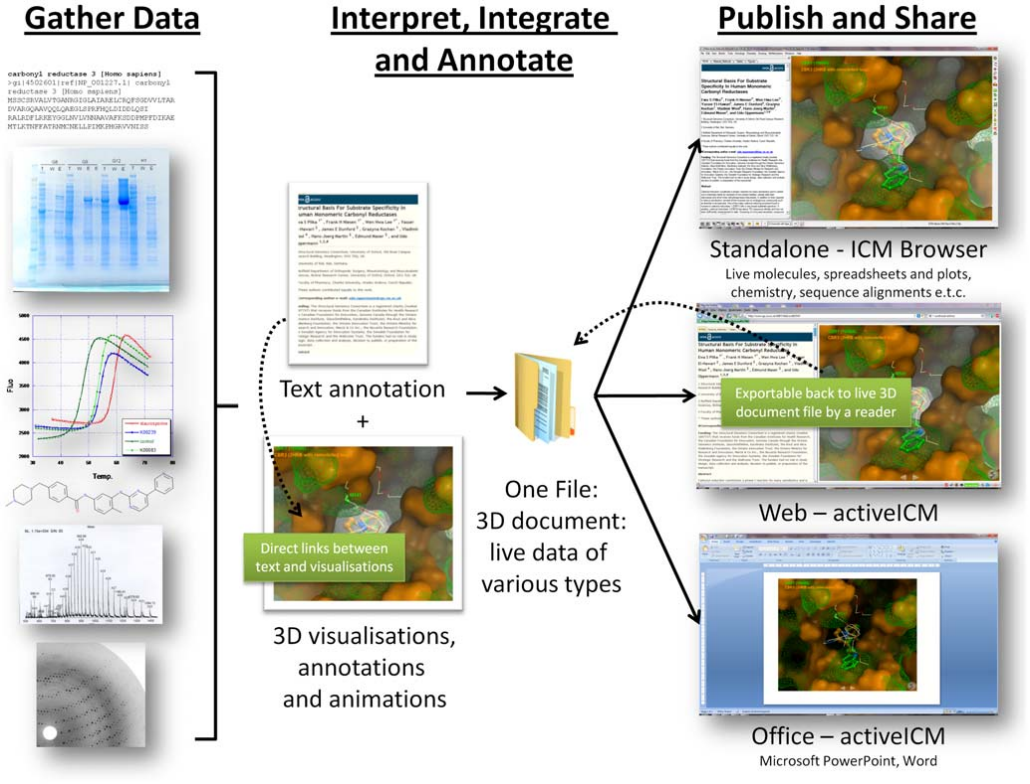
A workflow of scientific data generation and publication.

The elements of the proposed electronic publication method are shown in Figure 1. The Figure presents the principal steps, namely, (i) the integration of information into a single parsed binary file including multi-part documents, 3D objects, surfaces and labels; (ii) the serving of this file in three principle forms: a standalone single file form that does not require an internet connection, a form that is tuned for web delivery of the same content, and an Microsoft Office-embeddable form. The full standalone form of a 3D document can be browsed with a free multi-platform software package known as ICM Browser that also allows full extended access to additional data types, such as multiple sequence alignments, 2D chemical spreadsheets, tables and plots. The web form comprises of one or more web pages with JavaScript links to an embedded plugin, known as activeICM, for 3D visualization and can be generated automatically from the single standalone file. The web delivery of the 3D documents is focused towards the integrated visualization of 3D information in conjunction with cross-linked textual annotations. The Microsoft Office delivery uses an ActiveX wrapper around the activeICM technology to embed the 3D content with all views and animations into a standard PowerPoint or Word document.

The single 3D document and the possibility to export to web-delivered 3D content in this format provides a unique capability to easily distribute documents and electronic publications and to access them without direct web access in a platform-independent manner.

### Powerful and native graphics performance

Our method works on highly optimized and parsed native data structures representing the 3D objects that enables the display of large and complex objects in a highly detailed and nuanced manner. Latency, which may impact the usability and responsiveness of the visualization of 3D information, is minimized by in a number of methods. The data objects are already parsed and stored in a maximally compact single file. The file is automatically split into two or more files for the web delivery - one containing the text and visualizations and the other being a web page containing the text with links to the visualizations. This ensures that data is immediately accessible without any further processing being required because the visualization elements, viewpoints and views are already pre-calculated. The activeICM and ICM Browser software code, written in C and C++, has been highly pre-optimized for the native performance rather than optimized iteratively in real time at the expense of the initial graphics performance as is common in Java-based platforms.

### Multi-platform/multi-browser support

Supporting multiple platforms is essential as the scientific community is divided among the use of three main computer platforms, namely Windows, Mac OS/X and Linux. Despite the relative dominance of Windows in the general market, the market share of Mac OS/X and Linux in the research community is significant. Some communities generating 3D content are significantly more dedicated to non-Windows platforms. For example the crystallographic community or researchers using electron microscopy tend to use Linux as their primary platform for data analysis and visualization. Similarly, Mac OS X has been steadily gaining popularity among scientists. Only a small minority of free 3D visualization programs truly support multiple platforms, and only a fraction of those programs do so without any loss of functionality.

Web-browser plug-ins can be divided into two main types. The first plug-in type is an ActiveX component for Internet Explorer and Microsoft Office integration; the second plugin type needs to be written as Mozilla plug-in for Firefox and other compatible browsers such as Google Chrome and Safari. The plug-in code need to be further adjusted to a particular operating system. By supporting these two plug-in types, we cover almost 100% percent of the “browsing community.” Therefore we have implemented both solutions to be able to support multiple ways of the content delivery.

The technical challenges to supporting multiple platforms for both ICM Browser and activeICM are threefold: i) The operating system architecture. In spite of the OpenGL itself being cross-platform, there are many operating system dependent issues in terms of proper context creation and management inside the browser window. ii) Packaging and distribution. Each operating system has diverse approaches for packaging the files necessary for the software to function and even more diverse ways of capturing metadata associated with the software. For example, Windows requires registration of metadata in the registry database while Mac OS/X and Linux provide a completely different way of doing this. iii) Web browser heterogeneity. With multi-browser support, the main challenge is to deal with different application programming interfaces (APIs), which often have incompatible philosophies. For example there are major API differences between Internet Explorer and Mozilla plug-ins. Even within the second category the number of operating system-dependent operations is quite significant.

Whilst many of these issues are common to all software development projects, the result is the implicit requirement to dedicate resource to ensure that the functionality of the platform at least remains at a steady state.

### 3D figures or “slides”

A carefully prepared image of a molecular scene in a current printed or electronic publication becomes just a starting point for a live interactive 3D experience enhanced with animations and smooth transitions in the 3D articles. The central benefit of the 3D publication is the storage of a large number of different essential 3D visualizations (further referred to as 3D slides) along with the text and window layout. A 3D slide contains information about:

- a view point, e.g. rotation, translation, zoom, lighting and depth effects
- a set of atom-specific graphical representations such as surfaces, which can be represented as smooth, transparent or wireframe, ball-and-stick models, CPK (space-filling) models
- a set of atom, residue or distance labels on any of the atomic items
- a set of arbitrary 3D textual annotations assigned to a point in space
- a set of arbitrary 2D annotations assigned to specific 2D coordinates on a screen
- parameters of the parametric animation (see below).
- (for the standalone method of publishing with ICM Browser) parameters that define the position and types of windows or panels that are shown

Both 2D and 3D annotation can also possess multiple fonts, styles and colors. Most importantly, however, the slides possess the ability of rendering the molecular scene at arbitrary image resolution needed by a user, as well as modify the scene interactively for better rendition. They also come at minimal space penalty for the publication file due to the dynamic graphical rendition at reader's machine instead of downloading large high resolution images.

### Parametric animation

Animations give the viewer or reader a sense of context, but when a molecular movie is included in a PowerPoint file or web page there are a number of hurdles to be overcome: i) Loss of native resolution due to scaling of the document, and ii) Inability to directly interact with the animation until it is completed. For example, it is often not possible to change the view (e.g. zoom, translation) or even change representations on the fly. Critically, our 3D document animations are rendered on the fly thanks to the compact movement parameters associated with each transition. This means that the quality of the graphics is always optimal. Therefore the animations in the 3D slides are rendered at high resolution with no extra memory and file-space/bandwidth penalty compared to a pre-made movie and, critically, are interactive in terms of taking over the control of the view temporarily during an animation.

The animation types supported can be divided into three classes: i) a fixed type of movement around a current view, e.g. a sinusoidal rocking with different speed and amplitude and combinations of movements around X and Y axes, or rotations (with an infinite or fixed number of cycles); ii) conformational trajectories, illustrative or physical, with parametric interpolation. For example displaying a transition from one loop conformation to another using a few snapshots of intermediate states (see section ‘Showing conformation ensembles and transitions’ below); and iii) transition between arbitrary 3D slides (see below).

### Smooth transition between 3D slides

In a standard published 2D figure of a molecular structure, the spatial relationship between two figures is completely missing, not obvious, or intuitive. There may be some guidance of the spatial relationship between images such as the use of symbols to denote 180 or 90 degree rotations about an axis. However exploring a complex object interactively in 3D by providing a continuous animated path from one visualization to another provides a far more accessible and interpretive experience for the reader.

There are three types of transitions between two 3D slides within our 3D document: i) Making an abrupt change from one to another without any intermediate rendered trajectory frames; ii) Interpolation of the viewpoint between the 3D slides in a defined amount of time. On modern computers, the frame rate of even very complex visualizations is more then sufficient to show a smooth animation; and iii) Interpolation of the images by gradually blending them (fade in and out). This provides the opportunity to have one slide of (for example) a protein's active site and copy of this slide which shows a ligand and thereby fade in the ligand etc.

Typically we use method 2 and 3 sequentially using 500 ms each, but any combination of the methods can be chosen in practice.

### Linking text with 3D slides

An essential aspect of our approach is the integration of 3D slides with text. This allows an author of a 3D document to not only write about the data in question, but link the text written with 3D slides that the author also generates. The links are shown as standard hyperlinks within the text and when clicked upon cause the current visualization to transition to the required 3D slide. These links are defined through an easy to use graphical user interface.

When viewing a 3D document on a web site, the text is shown as HTML with the links to change the visualization in the activeICM plug-in being enabled via simple JavaScript commands.

### Full file exports for further browser or ICM use

The activeICM/web-based 3D document publishing method does require that the text component be split into an HTML file. This is possible via a ‘Save as…’ mechanism within the authoring platform ICM. Importantly, the accompanying ICM Browser file (.*icb*) still contains this text, enabling the further editing of the single .*icb* file without loss of fidelity.

### Showing electron density contours or NOE restraints with the structures

Historically, it has not been trivial to show electron density in the context of published macromolecular biological models, let alone for components of a structure. The use of 3D slide technology makes it possible for this feature to be used routinely in 3D publications. The method used in activeICM and ICM Browser makes it possible to easily download maps from the Uppsala Electron Density Server [2] and visualize and store contours of density of important parts of the structure (e.g. ligand) at virtually no space penalty. Similarly, in the structures determined by the Nuclear Magnetic Resonance, the NOEs and other restraints used for model building can be dynamically shown upon user request. The NOEs are treated as a special type of three dimensional distance or torsion labels.

### Showing conformation ensembles and transitions

For molecular objects, showing the available conformational space described by the experimental data (e.g. NMR) or simulation is essential because of the dynamic nature of those biological objects. Animations to show this, in contrast to simple discrete views, require more sophisticated techniques because intermediate conformations or frames must be calculated or interpolated for a smooth visualization. It is important that the transitions be performed with little penalty in terms of space and additional information required, i.e. with just a few parameters being required. The conformation transition requires additional data to generate those intermediate frames. Our concept consists of a number of key elements. Firstly, a small number of key intermediate frames is pre-generated and stored with the object. However those frames are not sufficient for a smooth, continuous, movement and can exist in either Cartesian or internal coordinate space. Secondly, depending on the nature of those reference frames, the procedure generates intermediate frames to provide the smooth animation. These interpolations can be derived in Cartesian, internal or mixed coordinate spaces. For example, the backbone of the polypeptide frame is interpolated in Cartesian space whilst side chains are interpolated in internal coordinate space. The intermediate frames in the internal coordinate space can provide a visual understanding of, for example, loop transitions in protein kinases or domain movements in which linkers undergo transitions and ligand:docking attempts. Both Cartesian and internal intermediate frames are stored in a very compressed manner - less than 10% of the size of the object per intermediate frame for Cartesian and around 1 to 2% smaller for internal coordinate intermediate frames.

### Image backgrounds

Molecular objects represent only a small part of a larger 3D environment. That scene can be enriched if a lightweight background image is displayed providing context for the underlying molecular action. The activeICM and ICM Browser are able to store and display any arbitrary image. For example, an image of a lipid bi-layer can be shown behind the structure of an integral membrane GPCR protein. Importantly, the background image is stored at the 3D slide level, meaning that a different background image can be used for each 3D slide.

### General 3D visualization and stereo

Whilst the background images for context is useful and has little space penalty, it is useful to provide the ability to display 3D and interactive non-atomic complex shapes with variable surface textures which move in concert with the atomic representations. To enable this display, the ICM Browser and activeICM supports the import of many popular 3D file formats. These include OBJ (Wavefront, http://local.wasp.uwa.edu.au/~pbourke/dataformats/obj), OFF (Object File Format, http://www.fileformat.info/format/off/egff.htm), KML (http://www.opengeospatial.org/standards/kml), DAE (Collada, http://www.collada.org) and 3DXML (http://www.3ds.com/products/3dvia/3d-xml/overview). An example where this is useful is to embed a integral membrane protein in the membrane of a cell, which is represented as a 3D shape with textures. The ICM browser also supports different types of stereo and can be directly used with the a stereo monitor of all major types.

### Exporting images

Being able to export the full document (as previously mentioned) for offline viewing and exchange is clearly important. However, the ability to export any visualization as a high resolution image from a 3D publication enables the reader to include this image in a traditional 2D publication as a figure or within a PowerPoint slide.

Our technology allows the level of detail to be increased depending on reader's computer and monitor capabilities. For example, the number of graphical triangles used to represent surfaces within a graphical primitive can be increased, resulting in a smoother surface. Anti-aliasing is also supported to provide hints for partially intersected pixels.

activeICM and ICM Browser are able to export images with larger dimensions than the screen via the use of tiling or a virtual screen. Tiling allows an arbitrary increase in the size of the exported image but is unable to support 3D labels. The use of a virtual screen resolves this issue and is limited only by available memory.

## Discussion

As the digital age marches on, it is important that the delivery of scientific information follows suit. It is time to consider and implement enhanced ways to present 3D information other than simple 2D static figures. Our 3D document technology transcends and helps to redefine the traditional methods of such publications, providing the ability to integrate and visualize 3D macromolecular information on the web and standalone in high detail whilst remaining interactive and intuitive to use.

We look forward to this approach taking hold in other areas of science and being adopted for electronic publishing.
